# A case report of syndrome of inappropriate antidiuretic hormone secretion unveiling hypothalamic involvement in multiple system atrophy

**DOI:** 10.3389/fendo.2026.1792679

**Published:** 2026-05-08

**Authors:** Zhe Long, Sheng Zeng

**Affiliations:** 1 Department of Neurology, The Second Xiangya Hospital, Central South University, Changsha, Hunan, China; 2 Clinical Medical Research Center for Stroke Prevention and Treatment of Hunan Province, Department of Neurology, The Second Xiangya Hospital, Central South University, Changsha, Hunan, China; 3 Department of Geriatrics, The Second Xiangya Hospital, Central South University, Changsha, Hunan, China

**Keywords:** central fever, multiple system atrophy (MSA), non-motor symptom, Shy–Drager syndrome, syndrome of inappropriate antidiuretic hormone secretion (SIADH)

## Abstract

Syndrome of inappropriate antidiuretic hormone secretion (SIADH) and central fever are rare and underrecognized manifestations of multiple system atrophy (MSA). Here, we report a 66-year-old woman who presented with a progressively evolving combination of parkinsonian features and pronounced autonomic dysfunction, fulfilling the diagnostic criteria for clinically probable MSA. Notably, she also developed two uncommon comorbidities: euvolemic hypotonic hyponatremia fulfilling the diagnostic criteria for SIADH, alongside episodic fever refractory to antipyretics in the absence of infection, but responsive to environmental cooling, consistent with central fever. These uncommon comorbidities underscore the involvement of hypothalamic and neuroendocrine regulatory pathways in MSA. This case broadens the well-recognized non-motor clinical spectrum of MSA and emphasizes the importance of considering central mechanisms when evaluating unexplained hyponatremia or fever in MSA. Awareness of these atypical manifestations may facilitate early recognition, guide targeted homeostatic management, and ultimately improve the quality of clinical care in MSA.

## 1 Introduction

Multiple system atrophy (MSA) is a rare neurodegenerative disease characterized by rapid progression and a poor prognosis (1, 2). Clinically, MSA patients present with a broad spectrum of motor and non−motor symptoms, encompassing several clinical syndromes—most prominently parkinsonism, cerebellar ataxia, and autonomic failure—any of which may occur in varying combinations and degrees (3). Two principal motor subtypes: parkinsonian variant (MSA−P) and cerebellar variant (MSA−C) were defined based on the updated diagnostic criteria in 2022 by the International Parkinson and Movement Disorders Society (2). Non-motor features, such as pure autonomic failure (e.g., genitourinary dysfunction, orthostatic hypotension) or isolated rapid eye movement (REM)-sleep behavior disorder, might emerge before motor symptoms onset (4, 5). Early autonomic failure has consistently been identified as a marker of poor prognosis (6, 7). For neuroimaging features, MSA is characterized by selective atrophy of the putamen, pons, and cerebellum, accompanied by increased diffusivity and abnormal iron deposition in the posterior putamen (1, 8). Specifically, the pontine hot−cross−bun (HCB) sign is a well−established and highly specific neuroimaging hallmark of MSA (9). As the disease advances, individuals with MSA typically experience increasing disability including unintelligible speech, severe dysphagia, frequent falls, wheelchair dependence, and eventual need for residential care, usually within 5.5 to 8 years after symptom onset (10).

Beyond the commonly recognized autonomic manifestations, endocrine and thermoregulatory disturbances are only rarely reported in MSA. Syndrome of inappropriate antidiuretic hormone secretion (SIADH) is an exceptionally uncommon non-motor feature, with only sporadic cases documented in the literature (11, 12). Notably, the co-occurrence of SIADH and central fever in MSA is exceedingly rare and poses a significant diagnostic challenge. This unique constellation of clinical presentations forms the distinctive feature of the present case, which we describe in detail below.

## 2 Case description

A 66-year-old woman (55 kg, 155 cm; body mass index 22.9 kg/m²) presented in August 2024 with a six−month history of dizziness and three-month history of bradykinesia, with noticeable symptom progression over the preceding two weeks. In February 2024, the patient developed dizziness characterized by a spinning sensation without blackout or syncope, accompanied by bilateral lower−limb joint and muscle pain, numbness, chest tightness, mild exertional dyspnea, and edema of the face and lower extremities. The diagnosis of “multiple lacunar infarctions” and “osteoporosis” were considered by a local hospital. Treatment with antiplatelet therapy, lipid−lowering agents, diuretics, and anti−osteoporotic medications led to resolution of edema, although dizziness, lower−limb weakness and muscle pain persisted. In May 2024, she experienced bradykinesia without an identifiable trigger. In July 2024, she was diagnosed with “parkinsonism” at a local hospital and started on treatment of carbidopa−levodopa (125mg three times daily) and pramipexole (0.125mg nightly), leading to mild symptomatic improvement. Brain magnetic resonance imaging (MRI), including T1-weighted imaging (T1-WI), T2-weighted imaging (T2-WI), susceptibility-weighted imaging (SWI), diffusion-weighted imaging (DWI), and magnetic resonance angiography (MRA), revealed multiple lacunar infarctions, leukoaraiosis and cerebral atrophy, cerebral arteriosclerosis, and symmetric hypointensity in the bilateral basal ganglia, likely representing physiological calcification. During the two weeks preceding the current admission, the patient experienced pronounced worsening of bradykinesia, lower−limb weakness and pain, and newly onset tremor when holding objects. She became unable to walk independently and experienced multiple falls without loss of consciousness. The patient was predominantly bedridden, with exacerbated bradyphrenia, hypophonia, and reduced facial expression. Since symptom onset, she experienced decreased appetite (approximately a two-thirds reduction from baseline), excessive daytime sleepiness, frequent nightmares, nocturia, urinary urgency with urge incontinence, while bowel habits have remained preserved. Regarding her past medical history, the patient has had a more than two-year history of urinary frequency and incomplete bladder emptying, with progressive worsening since February 2024, particularly nocturia. In addition, she underwent a cholecystectomy ten years ago and has a history of hypertension, which is managed with regular antihypertensive therapy. No family history of neurological diseases was reported.

On neurological examination, the patient remained alert and oriented, with intact memory and coherent responses. There was no nystagmus or diplopia, and extraocular movements were intact. Muscle strength was reduced, graded as 4- in lower limbs and 5- in upper limbs. Muscle tone was increased in all extremities with cogwheel rigidity. Finger-to-nose testing, rapid alternating movements, and heel-to-shin testing were bilaterally slow. The patient was unable to complete Romberg test, tandem gait, and pull test. Deep tendon reflexes were diffusely diminished.

Routine laboratory tests, liver function tests, myocardial enzyme profile, cardiac troponin, brain natriuretic peptide, serum ceruloplasmin, and circadian cortisol levels were within normal range. Decreased hemoglobin, creatinine, uric acid and free triiodothyronine (FT3) were detected, with free thyroxine (FT4) and thyroid-stimulating hormone (TSH) within normal range. Elevated fibrinogen and D−dimer levels were identified. Notably, a pronounced reduction in serum sodium (127.3mmol/L) accompanied by hypochloremia was identified, with 406 mOsm/kg urine osmolality. The 24-hour urinary sodium excretion was 170.82mmol/day. In conjunction with levels of serum potassium (4.17mmol/L), urea nitrogen (3.3mmol/L), and blood glucose (6.8mmol/L), resulting serum osmolality being 273.04 mOsm/kg, these findings indicated euvolemic hypotonic hyponatremia. Differential diagnoses for hyponatremia, including diuretic- or medication-induced hyponatremia, hypothyroidism, adrenal insufficiency, and cerebral salt wasting, were considered. However, the absence of relevant medication exposure, normal circadian cortisol levels, no biochemical evidence of hypothyroidism, and the lack of clinical signs of hypovolemia, together with inappropriately non-suppressed urinary sodium excretion despite hyponatremia, supported the diagnosis of SIADH rather than cerebral salt wasting. According to the Bartter–Schwartz criteria (13), the presence of hypotonic hyponatremia, inappropriately elevated urine osmolality, non-suppressed urinary sodium excretion, and clinical euvolemia established the diagnosis of SIADH in this patient. Plasma ADH levels were not measured due to limited availability in routine clinical practice.

Brain MRI demonstrated the pontine hot−cross−bun sign (**Figure 1A**) and right-sided putaminal rim sign (i.e., a hyperintense linear signal at the lateral border of the putamen) were detected in T2-WI imaging (**Figure 1B**). Brain positron emission tomography computed tomography (PET-CT), including dopamine transporter (DAT) and glucose metabolism (FDG) imaging, showed markedly reduced dopamine transporter uptake in the bilateral striatum (most pronounced in the putamen) (**Figure 2A**), with mildly decreased glucose metabolism in the striatum (predominantly posterior putamen and caudate) (**Figures 2B, C**). Additional neuroimaging findings included microhemorrhage in the right cerebellar hemisphere, cerebral arteriosclerosis, segmental stenosis of the left posterior cerebral artery (PCA), and calcifications in the bilateral basal ganglia.

**Figure 1:**
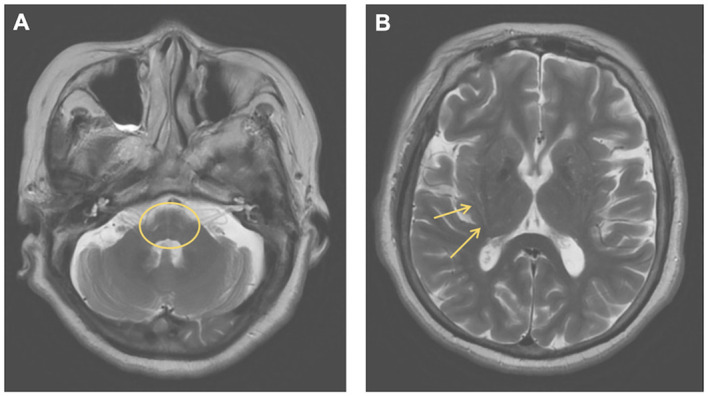
The brain MRI findings in the patient. **(A)** The characteristic hot−cross−bun sign was illustrated in pons in T2-weighted imaging (T2-WI). **(B)** A putaminal rim sign was identified in T2-WI, appearing as a hyperintense linear signal along the lateral margin of the right putamen.

**Figure 2:**
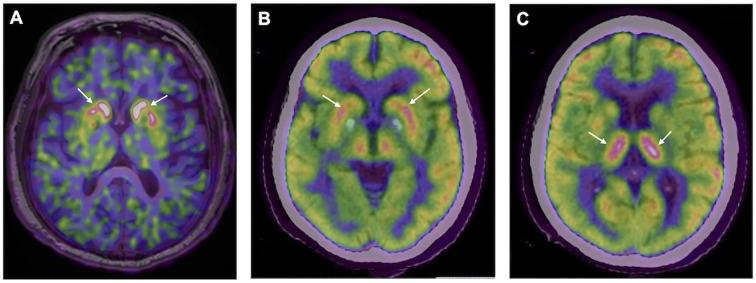
The PET-CT features in the patient. **(A)** Dopamine transporter (DAT) imaging showed markedly reduced dopamine transporter uptake in the bilateral striatum (most pronounced in the putamen). **(B, C)** Glucose metabolism (FDG) imaging illustrated mildly decreased glucose metabolism in the striatum (predominantly posterior putamen and caudate).

Orthostatic hypotension (OH) was documented. Blood pressure was 121/80 mmHg with heart rate of 63 beats per minute in the supine position; after 3 minutes of standing, the blood pressure fell to 82/58 mmHg with a heart rate of 69 beats per minute, consistent with neurogenic orthostatic hypotension. In addition, 24-hour ambulatory blood pressure monitoring presented that daytime blood pressure values were within normal range, with an mean systolic and diastolic pressures of 117 mmHg and 76 mmHg, respectively. During nighttime, mean systolic and diastolic pressures increased to 137 mmHg and 82 mmHg, respectively, fulfill with nocturnal hypertension. Other examinations revealed a post−void residual urine volume of approximately 22 mL. Lumbar spine MRI showed a new compression fracture of the L2 vertebral body, along with degenerative lumbar spine changes and disc bulging at L3/4, L4/5, and L5/S1 levels. Decreased bone mineral density was identified in lumbar spine, left femoral neck and left hip. Transthoracic echocardiography revealed degenerative changes of the aortic valve with mild regurgitation, while left ventricular systolic function remained within the normal range. Lower−limb vascular ultrasound detected thrombosis in the intramuscular veins of both calves. Chest and abdominal CT demonstrated scattered pulmonary micronodules, classified as lung imaging reporting and data system (LU−RADS) category 2, mild enlargement of the left hepatic lobe and a small right renal calculus.

During hospitalization, the patient developed a fever with a peak temperature of 38.9 °C, measured using a mercury axillary thermometer. Body temperature was routinely monitored three times daily. The febrile episode lasted approximately two days. When the body temperature exceeded 38.5 °C, oral ibuprofen suspension (10 mL, equivalent to 200 mg) was administered. Notably, only one episode of high-grade fever (38.9 °C) occurred, and no significant reduction in temperature was observed following antipyretic administration. When the body temperature was below 38.5 °C, physical cooling measures, including the application of ice packs, were implemented. At the time of the fever episode, the patient exhibited anhidrosis. Urgent laboratory tests, including complete blood count, C−reactive protein, erythrocyte sedimentation rate, procalcitonin, PCR testing for COVID−19 and influenza A/B, were all within normal limits. The ambient room temperature during hospitalization was maintained at approximately 28 °C. After exclusion of infectious and inflammatory causes, active environmental cooling was initiated by lowering the room temperature to approximately 20–22 °C. Following this intervention, her fever gradually subsided and did not recur (**Figure 3A**). Importantly, the resolution of fever occurred only after environmental cooling rather than pharmacological treatment. At the time of the febrile episode, bradycardia was not observed. Serum sodium levels during the fever ranged from 127.3 to 128.8 mmol/L. For the management of SIADH, fluid restriction (≤1000 mL/day) and oral supplementation with 10% sodium chloride solution (20 mL daily) were initiated. Subsequently, serum sodium levels gradually returned to normal (**Figure 3B**). These findings were suggestive of central fever. Differential diagnoses for fever, including infectious etiologies (e.g., pneumonia or viral infection), drug-induced fever, and systemic inflammatory conditions, were carefully evaluated. The absence of infectious symptoms, normal inflammatory markers, negative viral PCR testing, presence of anhidrosis, the lack of response to antipyretics but rapid resolution with environmental cooling and the potential background of autonomic dysfunction in MSA supported a central origin. These features are consistent with impaired thermoregulatory control observed in autonomic failure, as described in the Mayo Clinic autonomic disorder framework, which characterizes abnormalities such as reduced sweating, impaired heat dissipation, and altered peripheral thermoregulation (2, 14). Documentation of peripheral skin temperature (e.g., cool extremities) was not available, and no overt heat intolerance was reported under standard ambient conditions.

**Figure 3:**
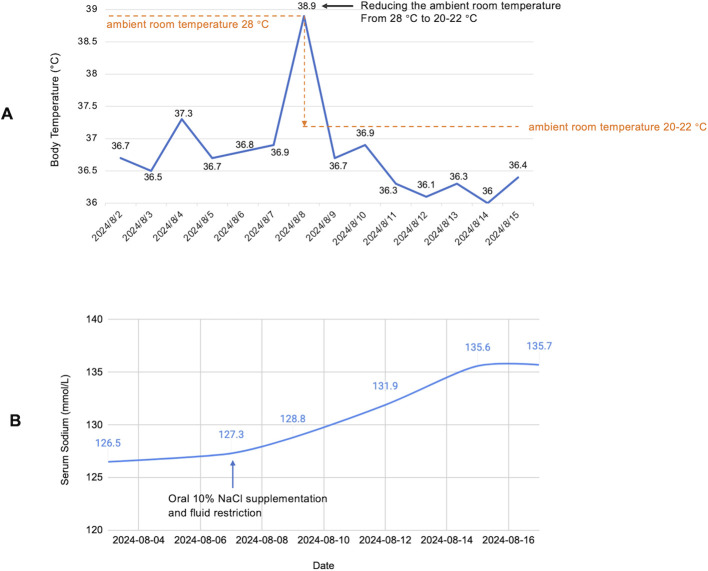
**(A)** Temporal profile of body temperature and identification of central fever. The line graph illustrates daily body temperature fluctuations during hospitalization, measured using an axillary thermometer three times daily. The patient developed a febrile episode lasting approximately two days, with a peak temperature of 38.9 °C. When the body temperature exceeded 38.5 °C, oral ibuprofen suspension (10 mL, equivalent to 200 mg) was administered, with no significant reduction in temperature observed. Physical cooling measures (e.g., ice packs) were applied when the temperature was below 38.5 °C. The ambient room temperature was initially maintained at approximately 28 °C (orange dotted line) and was subsequently reduced to 20–22 °C (arrow, orange dotted line), after which the fever gradually subsided and did not recur. The episode was further characterized by anhidrosis and the absence of infectious or inflammatory findings. The resolution of fever following environmental cooling rather than pharmacological intervention supported the diagnosis of central fever. Subsequent monitoring showed stabilization of the temperature within the normal range. **(B)** Longitudinal trend of serum sodium levels and clinical intervention. Upon admission, the patient exhibited significant hyponatremia with a baseline level of 126.5 mmol/L. Following the diagnosis of the syndrome of inappropriate antidiuresis (SIADH), therapeutic interventions was conducted on August 7, 2024, when serum sodium was 127.3 mmol/L (arrow), including fluid restriction (≤1000 mL/day) and oral supplementation with 10% sodium chloride solution (20 mL daily). A steady increase was observed thereafter, serum sodium and chloride levels gradually returned to normal range. The X axis showed the date and Y axis illustrated serum sodium (mmol/L).

During hospitalization, treatment with carbidopa-levodopa was 125mg three times daily, and pramipexole was increased to 0.125mg three times daily. Selegiline (5mg once daily) was additionally initiated. Notably, previously reported cases of hyperthermia in MSA were frequently associated with concomitant antiparkinsonian medications. In contrast, although our patient was receiving dopaminergic therapy, the clinical features—particularly anhidrosis and the clear response to environmental cooling rather than pharmacological intervention—support a central thermoregulatory dysfunction rather than drug-induced hyperthermia. The patient reported a slight improvement in dizziness during the inpatient period; however, motor symptoms showed limited responsiveness to dopaminergic therapy. As part of non-pharmacological management, the patient and family were advised to avoid high ambient temperatures, maintain a controlled ambient environment, and monitor body temperature regularly. Patient education, lifestyle adjustments, waist-high stockings and elevate head of bed were performed for orthostatic hypotension. Pelvic floor training and timed voiding were conducted for urinary urgency and urge incontinence. Following discharge, the patient returned to a rural home setting and did not undergo regular outpatient follow-up or institutional care. Although partial improvement of hyponatremia and fever was achieved at discharge, adherence to recommended monitoring and management was limited. As a result, longitudinal data on serum sodium levels, orthostatic hypotension, and recurrent febrile episodes were not available. Repeated telephone follow-up revealed continued progression of bradykinesia and overall functional decline, with the patient becoming predominantly bedridden. The patient passed away in May 2025; however, the exact cause of death could not be ascertained, as the family declined to provide further details. Given the advanced disease stage at presentation, rapid clinical progression, and prominent autonomic dysfunction, a poor prognosis was anticipated. In addition, limited access to ongoing medical care and suboptimal adherence to follow-up may have further contributed to the unfavorable outcome.

## 3 Discussion

This 66-year old female patient presented with a progressively clinical coexistence of parkinsonism features (e.g., bradykinesia, cogwheel rigidity and reduced facial expression) and profound autonomic dysfunction, manifested as neurogenic orthostatic hypotension, nocturnal hypertension, urinary urgency and urge incontinence. Brain structural MRI demonstrated multiple hallmark features of MSA, including the hot-cross-bun sign in the pons and putaminal rim sign. Furthermore, dopamine transporter PET revealed markedly reduced striatal dopamine transporter uptake, predominantly in the putamen, consistent with presynaptic dopaminergic degeneration, which is a characteristic feature of MSA (15, 16). Taken together, these clinical and neuroimaging findings fulfill the diagnostic criteria for clinically probable MSA (2).

Notably, in addition to typical motor and autonomic manifestations, this patient also developed concurrent SIADH and central fever. The patient exhibited euvolemic hypotonic hyponatremia, with low serum sodium and osmolality in the presence of inappropriately normal urinary sodium excretion, fulfilling the essential diagnostic criteria for SIADH (17). Additionally, she experienced an episode of high fever with anhidrosis and lack of responsive to antipyretics, without evidence of infection or systemic inflammation, the fever resolved with environmental temperature cooling, supporting central fever diagnosis (18). Taken together, this report described an elderly woman with clinically probable MSA-P who illustrated the rare comorbid occurrence of SIADH and central fever, two rare and underrecognized non-motor manifestations in MSA. This case broadens the recognized MSA clinical spectrum and underscores the potential involvement of central neuroendocrine and thermoregulatory networks.

Hyponatremia in MSA is uncommon, and coexistence of MSA and SIADH has been documented only in isolated case reports and small case series (**Table 1**) (11, 12). Previous reports have suggested that SIADH in MSA was attributable to MSA process itself and to comorbid conditions such as pneumonia, rather than to antiparkinsonian medications (12). MSA, similar to other neurodegenerative disorders, can affect the hypothalamus (19, 20), which functions as the primary regulatory hub for fluid homeostasis. The supraoptic and paraventricular nuclei of the hypothalamus synthesize antidiuretic hormone (ADH) (21), which is subsequently transported to the posterior pituitary for storage and release into the circulation to maintain osmotic equilibrium (22). Beyond its primary role in osmoregulation and sodium homeostasis, ADH is integral to systemic hemodynamics and renal function (23). α-synuclein inclusions and neuronal loss were identified in the supraoptic and paraventricular nuclei of the hypothalamus in MSA (19, 24), providing a structural substrate for impaired ADH regulation that may lead to SIADH and dilutional hyponatremia. Although rare, SIADH may be under−recognized, particularly in MSA patients with advanced autonomic dysfunction. The present case reinforces that disturbances in water–electrolyte balance may be an intrinsic and biologically plausible manifestation of MSA.

**Table 1:** The summary clinical information of patients with MSA and SIADH.

Patient	Age (y)	Sex	Duration (y)	MSA type	Na (mmol/L)	K (mmol/L)	Cl (mmol/L)	ADH (pg/mL)	Urine Osm (mOsm/kg)	Serum Osm (mOsm/kg)	24-H urine Na (mmol/day)	Concomitant diseases	Antiparkinson drugs
1	66	F	2	P	127.3	4.17	89.6	–	406	273.04	170.82	Central fever	Yes
2	54	M	2	P	123	3.9	92	–	989	252	338	Pneumonia	Yes
3	63	M	2	P	118	–	–	–	479	267	108	Pneumonia	No
4	53	M	2	C	122	–	–	–	945	258	209	Pneumonia	No
5	62	M	3	P	123	–	–	–	457	280	91	Pneumonia	No
6	59	M	4	C	118	–	–	–	–	–	144	Pneumonia	No
7	53	M	5	P	121	–	–	–	–	–	129	–	Yes
8	52	M	4	P	127	4.2	96	3.5	319	262	–	–	Yes
9	61	F	9	C	99	3.7	66	25.5	474	205	–	Pneumonia	Yes
10	67	M	11	P	124	5.5	89	1.48	420	255	–	Pacemaker	Yes
11*	52	M	10	C	120	–	–	2.1	381	246	–	Fever	Yes
12	76	M	1.5	P	118	4	84	3.5	304	244	–	–	No
13	62	F	8	C	118	–	66	12	–	254	–	Sleep Apnea	No
14	58	F	6	P	116	–	–	11.7	722	–	–	Pyelonephritis	Yes

1. Patient No.1 refers to the patient in this case report. Patient No.2 indicates the case in reference (27) (in Chinese). Patient No.3 shows the case in reference (28) (in Chinese). Patient No.4 indicates the case in reference (29) (in Chinese). Patient No.5 to 7 reveal the cases in reference (30) (in Chinese). Patient No.8 to 14 indicates the case in reference (12).

2. Plasma ADH levels were not measured. The diagnosis of SIADH was established based on fulfillment of the Bartter–Schwartz criteria.

*Central fever was not explicitly reported in the previously published cases. In case No. 11, only “fever” was mentioned in the secondary source, without sufficient detail to determine whether it represented central fever.

The co-occurrence of central fever in this patient further highlights the involvement of hypothalamic and brainstem thermoregulatory pathways. Central fever is typically defined by elevated body temperature without infection, inflammation, or response to antipyretic medications, and is commonly observed in acute brain injury or neurodegenerative conditions that disrupt thermoregulatory centers. Thermoregulation is primarily regulated by the preoptic and posterior hypothalamic nuclei of the hypothalamus (21), which integrate afferent thermal signals from the brainstem, spinal cord, and peripheral thermoreceptors (25). Degeneration within these structures may alter the hypothalamic temperature set point, resulting in dysregulated heat production and impaired heat dissipation. In MSA, pathological involvement of the hypothalamus and autonomic brainstem nuclei has been well documented (24), providing a plausible mechanism for the development of central fever in rare cases. The anhidrosis observed in this patient further supports autonomic failure contributing to impaired heat loss.

Potential triggers of central fever in MSA include progressive hypothalamic and brainstem degeneration and environmental stressors, such as elevated ambient temperature, which may precipitate fever episodes in this context. Prognostically, central fever episodes may resolve with supportive measures, but their occurrence reflects advanced hypothalamic-autonomic involvement and may signal a more complex disease trajectory. Notably, MSA patients presenting with prominent autonomic dysfunction as an early clinical manifestation may exhibit a more aggressive disease course and poorer prognosis, which is consistent with the rapid progression observed in the present case. Notably, in the classical description of Shy–Drager syndrome—now recognized as part of the multiple system atrophy (MSA) spectrum with predominant autonomic failure—severe autonomic dysfunction has been associated with a more aggressive disease course and poorer prognosis. In contemporary clinical practice, although MSA is primarily classified into parkinsonian (MSA-P) and cerebellar (MSA-C) subtypes, autonomic dysfunction remains a core feature across phenotypes (1). In this context, the rapid progression observed in our patient—characterized by early and prominent autonomic dysfunction, together with the coexistence of SIADH and central fever—may reflect extensive involvement of hypothalamic and brainstem regulatory centers.

From a network-based perspective, MSA is a multisystem neurodegenerative disorder affecting interconnected circuits across the basal ganglia, cerebellum, brainstem, and hypothalamus. While motor phenotype arises from striatonigral and olivopontocerebellar degeneration, non-motor manifestations such as autonomic dysfunction, SIADH, and central fever likely reflect disruption of hypothalamic-brainstem-pituitary networks. The simultaneous occurrence of SIADH and central fever in this patient suggests a shared pathological substrate involving hypothalamic dysfunction. This observation reinforces the evolving concept that MSA extends beyond motor and classical autonomic pathways to encompass broader neuroendocrine and homeostatic systems (26).

This case provides several significant clinical insights. First, SIADH and central fever could be considered as potential, though rare, manifestations of MSA disease spectrum, particularly in patients with advanced or rapidly progressive autonomic dysfunction. Unexplained hyponatremia or hyperthermia in MSA should arise consideration of central homeostatic dysregulation, rather than being attributed exclusively to infection, pharmacotherapy, or systemic comorbidities. Early recognition of SIADH allows timely management with fluid restriction and sodium supplementation, potentially preventing neurological deterioration, such as exacerbating gait instability, bradyphrenia and an increased risk of falls. Second, central fever in MSA may be refractory to conventional antipyretics, and environmental temperature cooling as well as physical temperature regulation may be more effective therapeutic strategies than traditional pharmacological approaches. From a research perspective, this case highlights the need for further studies exploring the prevalence and diagnostic markers of hypothalamic dysfunction in MSA. Future investigation using neuroimaging, detailed neuropathological analysis, and targeted neuroendocrine assays are useful approaches to map the extent of hypothalamic involvement. A deeper understanding of these underlying mechanisms may open avenues for more precise symptomatic therapies and significantly enhance the management of non-motor complications in MSA.

Unfortunately, the patient passed away before formal patient-reported outcomes could be collected; however, her family expressed satisfaction with the improvement in hyponatremia and fever.

## 4 Conclusion

In summary, we report a patient with clinically probable MSA-P complicated by SIADH and central fever, two rare and underrecognized manifestations of the disease. This case expands the well-known clinical spectrum of MSA and highlights the contribution of hypothalamic and neuroendocrine dysfunction in its pathophysiology. Awareness of these atypical features may support earlier recognition, guide appropriate management, and ultimately improve care for MSA patients.

## Data Availability

The original contributions presented in the study are included in the article/supplementary material. Further inquiries can be directed to the corresponding author.
